# Antimicrobial Usage Among Different Age Categories and Herd Sizes in Swiss Farrow-to-Finish Farms

**DOI:** 10.3389/fvets.2020.566529

**Published:** 2020-12-15

**Authors:** Thomas Echtermann, Cedric Muentener, Xaver Sidler, Dolf Kuemmerlen

**Affiliations:** ^1^Division of Swine Medicine, Department for Farm Animals, Vetsuisse Faculty, University of Zurich, Zurich, Switzerland; ^2^Institute of Veterinary Pharmacology and Toxicology, Vetsuisse Faculty, University of Zurich, Zurich, Switzerland

**Keywords:** antimicrobial drug usage, defined daily dose, age category, herd size, monitoring systems, pigs, Switzerland

## Abstract

In the Swiss pig sector, the usage of antimicrobials has been recorded, evaluated and systematically reduced on a voluntary basis since 2015. This monitoring has been carried out using various methods thereby enabling continuous national scrutiny as well as international comparisons. To gain a better understanding of the dynamics of the antimicrobial usage on Swiss farms, consumption data of farrow-to-finish farms were analyzed for (i) the within-herd relationships among different age categories and (ii) the influence of the herd size. The data were collected on 71 farms for the year 2017, encompassing the amount of active ingredients and number of defined daily doses Switzerland (nDDDch) in total, and stratified for the different age categories of piglets, weaners, fattening pigs, and sows. The differences in nDDDch per animal among the age categories were determined by a Wilcoxon test and subsequent *post-hoc* analysis according to Bonferroni. The within-herd relationship among the individual age categories as well as the influence of the herd size on nDDDch per animal measured as kept sows were analyzed by simple linear regression. The evaluation of the treatment days showed that 50% of the nDDDch were used in piglets, 44% for weaners, and 3% each for fattening pigs and sows. Compared to the other age categories, the examination of the number of nDDDch per animal showed a significantly higher number for sows, whereas for fattening pigs the number was significantly lower (*P* < 0.01). The farm-based analysis using linear regression showed a relationship between antimicrobial usage in sows and piglets (*P* < 0.001; adj. *R*^2^ = 0.19). Similarly, a significant relationship between larger herd size and increased antimicrobial usage was observed (*P* = 0.02; adj. *R*^2^ = 0.06). The present study provides an insight into the antimicrobial treatment dynamics of farrow-to-finish farms. In particular, the age categories piglets and sows—with their higher number of treatment days in total or per animal—are of interest regarding the potential reduction in antimicrobial usage. Likewise, larger farms with higher management requirements were found to be of particular importance for the reduction of antimicrobial usage. Monitoring programs should therefore evaluate different age categories separately to identify problems for individual farms.

## Introduction

Awareness of the spread of antimicrobial-resistant genes is an important issue at the public health interface between human and veterinary medicine (1). The general transfer of these genes between both disciplines is well documented (2–4). One of the most important drivers to reduce this spread is optimized and prudent antimicrobial drug usage (AMU) (5). This approach of careful usage is consistently demanded for our farm animals and is also reflected by the report of the European Surveillance of Veterinary Antimicrobial Consumption (ESVAC), where Switzerland performs moderately within a European context (6). In addition to the guidelines of the World Health Organization (WHO) (7), countries like Switzerland have therefore published guidelines describing the evidence-based usage of antimicrobials for the important livestock species cattle and pigs (8).

In addition to treatment guidelines, the first important step is setting up a powerful AMU monitoring system, which allows for correlations with resistance data (9) and is linked to animal performance and certain aspects of biosecurity and prevention (10–13). Such monitoring systems may be based on several different measurement methodologies, which, although valid and reliable by themselves, could nevertheless complicate comparisons among different regions and species (14). Such differences in the calculation must be taken into account. An established and common monitoring strategy is based on the measurement of so-called defined daily doses, which allow for the estimation of the potential number of treatment days from the used amount of antimicrobial ingredients (15, 16). This measurement method, originally developed for human medicine (17), was adopted in veterinary medicine and examined in several studies monitoring the AMU in pigs (18, 19). Furthermore, the European Medicines Agency (EMA) developed a detailed description for the assignment of defined daily doses and treatment course doses in animals (20), supplemented by values for defined daily doses (DDDvet) and defined course doses (DCDvet) for the livestock sector (21).

According to this description, nationally defined doses (DDDch/DCDch) were developed for the pig sector in Switzerland and compared with the values of the EMA in the field (22, 23). These defined doses of Switzerland were used in a further study to determine the usage of Highest Priority Critically Important Antimicrobials according to WHO (HPCIAs) and the Swiss Federal Ordinance on veterinary medicines as well as to investigate the association between AMU and different types of farms (24, 25).

In addition to comparing different types of farms, the relationship among AMU and different age categories within farrow-to-finish farms has also been examined and described with other monitoring systems (26). This study found a positive relationship among the AMU within the studied age categories, so the AMU in one age category was positive related to the AMU in another age category on the farm. The influence of larger herd size on increasing antibiotic consumption has also been investigated by several authors using different monitoring strategies, with some studies observed (27, 28) while other studies found only weak correlations (29) or not observed (30), such a relationship.

The objectives of this study were (1) to investigate the association among AMU and animal age category on farrow-to-finish farms and (2) to investigate the association between AMU and herd size on farrow-to-finish farms. The hypotheses of this study were that (1) AMU would differ among age categories on farrow-to-finish farms, and (2) AMU would differ among herd sizes on farrow-to-finish farms. AMU was measured using DDDch, and herd size was measured in the number of sows kept.

## Materials and Methods

### Data Collection

In collaboration with the Swiss Pig Health Service (SSHS), AMU data were collected from 71 Swiss farrow-to-finish pig farms in 2017. The study farms joined a nationwide voluntary program known as Suissano/Safety+, which evaluates the AMU on their farms to improve transparency along the pig production process. Overall, 598 out of 6,406 pig farms throughout Switzerland participated in the Suissano/Safety+ program in 2017, which represents a proportion of 9% (31). Out of the 598 farms, 71 farms were defined as farrow-to-finish by the SSHS as farms housing at least 10% of the piglets from birth until the time of slaughter. The farms were independent of each other and not part of a larger system. The distribution of the study farms corresponds to the distribution of Swiss pig production with its higher animal populations in the cantons of Lucerne and St. Gallen. Only farms with complete documentation for antimicrobial preparations purchased in 2017 were included in the study. According to the Swiss Federal Ordinance on veterinary medicines, purchased preparations containing antimicrobial ingredients must be used within a maximum time period of 3 months (25). The study farms were required to document all veterinary prescriptions of antimicrobial ingredients for that year, including exact information on the name and quantity of the products used. Supported by the herd veterinarian and the SSHS, farmers had to allocate the prescribed antimicrobial agents to four different categories (piglets, weaners, fatteners, and sows). The age categories were defined in such a way that a piglet was counted from birth until the end of the 4th week of life, a weaner from the 5th week of life until the end of the 12th week of life, and a fattening pig from the 13th week of life until slaughter. Gilts and boars were counted as sows from the age of 7 months. This classification was communicated by the SSHS when a farm was included in the Suissano/Safety+ program. In addition to the AMU records, the number of pigs kept (sows) or produced annually (all other age groups) were recorded.

### AMU Quantification

To quantify AMU, the amount of prescribed antimicrobial ingredient from the study farms during 2017 was divided by the defined daily doses Switzerland (DDDch) of each corresponding antimicrobial classes multiplied by the standard weights of the different age categories as defined by the European Surveillance of Veterinary Antimicrobial Consumption (ESVAC) (piglets: 4 kg; weaners: 12 kg; and fatteners: 50 kg and sows: 220 kg) (32).

$$\begin{array}{l} Number\;of\;defined\;daily\;doses\;Switzerland\;\left(nDDDch\right)\\ =\frac{\sum total\;amount\;ofprescribed\;antimicrobial\;ingredient\;\left(mg\right)}{DDDch\;\left(\frac{mg}{kg}\right)\;*\;standard\;weight\;of\;age\;category\;\left(kg\right)} \end{array}$$

The required information describing the doses in mg/kg was taken from the product leaflets which are available in the Swiss Veterinary Medicines Drug Compendium (33). The detailed procedure for defining the national doses and all values for DDDch have been published in previous work from our research group (22).

The number of DDDch (nDDDch) and the amount of prescribed antimicrobial ingredients were calculated in total, as were the different age categories and the different used antimicrobial classes.

For the evaluation of the AMU among different herd sizes and to compare the consumption by different categories, the number of kept (sows) or produced pigs (piglets, weaners, and fatteners) in the year 2017 were considered according to the following formula:

$$\begin{array}{l} nDDDch/animal/year=\frac{\sum total\;amount\;of\;prescribed\;antimicrobial\;ingredient\;per\;farm\;and\;age\;category\;\left(mg\right)}{DDDch\;\left(\frac{mg}{kg}\right)\;*\;standard\;weight\;of\;age\;category\;\left(kg\right)\;*\;number\;of\;pigs\;per\;farm\;and\;age\;category} \end{array}$$

This calculation was performed separately for each prescribed antimicrobial ingredient used on the farm, and the resulting numbers were added up for each age category. Finally, the results of the different age categories were summarized as the total nDDDch per pig in 2017. The herd size was defined as the number of sows kept.

### Data Processing and Statistical Analysis

The preparation of all operating farm data and the calculation of the number of defined doses were carried out using Microsoft Excel 2016 (Microsoft, Redmond, WA, USA). The statistical analysis and preparation of graphs to visualize the results were performed with R (https://cran.r-project.org). Differences among the tested groups having a *P* ≤ 0.05 were considered statistically significant. The data for calculated AMU on the farm level were tested for normal distribution and the differences among the individual age categories were investigated using the Kruskal-Wallis test followed by a *post-hoc* pairwise analysis (Bonferroni correction). The relationship between the herd sizes and the nDDDch/animal/year on farms was examined by a simple linear regression. Both were set as continuous variables with herd size as the predictor and nDDDch/animal/year as the response variable. Similarly, simple linear regression was used to compare the nDDDch/animal/year for the different age categories. The nDDDch/animal/year of sows or the respective younger age category was determined as the predictor variable and was examined with the nDDDch/animal/year of another age category as the response variable. The nDDDch/animal/year of the six different models of predictor-response variables (sows-piglets, sows-weaners, sows-fatteners, piglets-weaners, piglets-fatteners, and weaners-fatteners) was therefore analyzed. Homoscedasticity tests and plots were examined to check whether assumptions of normality and homoscedasticity were fulfilled and scatterplots were prepared to visualize the results.

## Results

### AMU Quantification per Age Category

The total and relative distribution of the antimicrobial consumption within different age categories measured as nDDDch as well as the active antimicrobial ingredient is given in Table 1. In terms of AMU monitoring calculated as nDDDch, piglets (50%) and weaners (44%) were the most frequently observed age categories, while weaners (44%), and sows (36%) were the most frequently observed age categories for administered active ingredients. The antimicrobial consumption in fatteners was 18,399 nDDDch (3%) and 9.2 kg of active ingredient (9%), respectively.

**Table 1:** Total and relative (%) distribution of antimicrobial consumption within the different age categories (piglets, weaners, fatteners, and sows) measured either as number of defined daily doses Switzerland (nDDDch) or as active antimicrobial ingredients.

	**nDDDch**	**%**	**Active ingredient in kg**	**%**
Piglets	314,743	50%	9.8	10%
Weaners	276,038	44%	44.0	44%
Fatteners	18,399	3%	9.2	9%
Sows	17,799	3%	36.1	36%
Overall	626,979		99.1	

**Figure 1:**
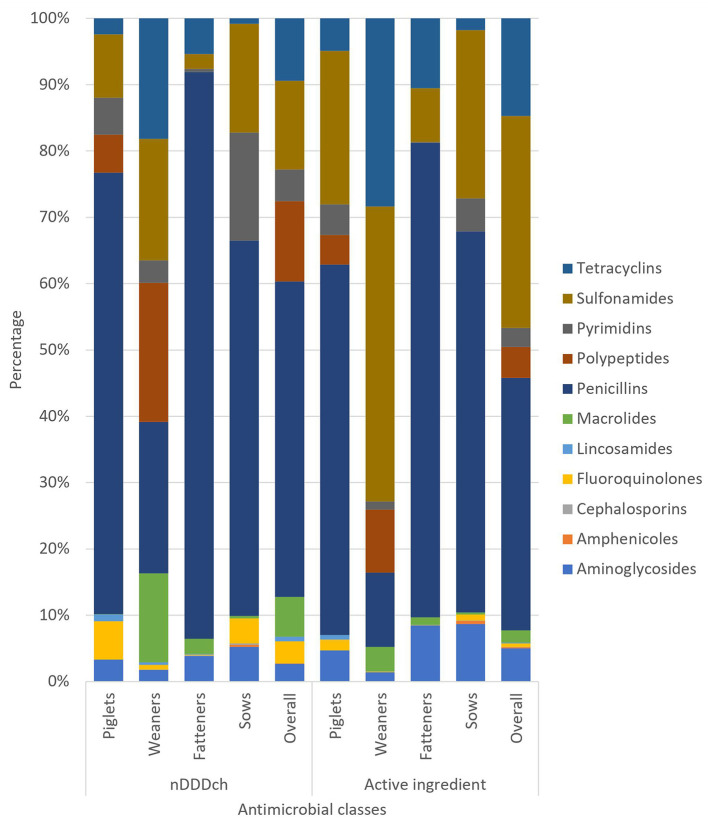
Relative distribution of the used antimicrobial classes analyzed either as nDDDch or as active ingredient of the overall study population and separated among the different age categories (piglets, weaners, fatteners, and sows).

The overall distribution as well as the distribution within the different age categories of the used antimicrobial classes analyzed either as nDDDch or as active ingredient are shown in Figure 1. Penicillins were the most frequently observed used antimicrobial classes overall with 298,311 nDDDch (48%) and 37.8 kg (38%) measured active ingredients, respectively. Apart from the measured active ingredients in weaners, penicillins were also the most frequently observed antimicrobial class in the different age categories. For the weaners, 19.6 kg (44%) sulfonamides and 12.5 kg (28%) tetracyclines were observed when measuring active ingredients. Analyzing the HPCIAs cephalosporins, fluoroquinolones, macrolides, and polypeptides, the overall proportions of these antimicrobial classes were 21% for the calculated nDDDch results and 7% for the amount of active ingredients, respectively.

Table 2 summarizes the descriptive statistics for the distribution of the number of animals per farm. These herd size numbers for the different age categories were included for each individual farm to calculate the number of DDDch per animal in the year 2017.

**Table 2:** Descriptive statistics on the distribution of different age categories on the study farms measured as number of pigs kept (sows) and number of produced pigs per year (piglets, weaners, and fatteners) on 71 farrow-to-finish pig farms in Switzerland in 2017.

**Age category**	**Mean**	**Median**	**CI (95%)**	**SD**	**Min**	**Max**
Piglets	2,390	2238	2,085–2,694	1,267	359	6,040
Weaners	2,061	1,918	1,803–2,319	1,075	320	5,200
Fatteners	936	790	750–1,122	775	15	3,800
Sows	79	74	69–88	43	10	220

*CI, confidence interval; SD, standard deviation; Min, minimum; Max, maximum*.

**Figure 2:**
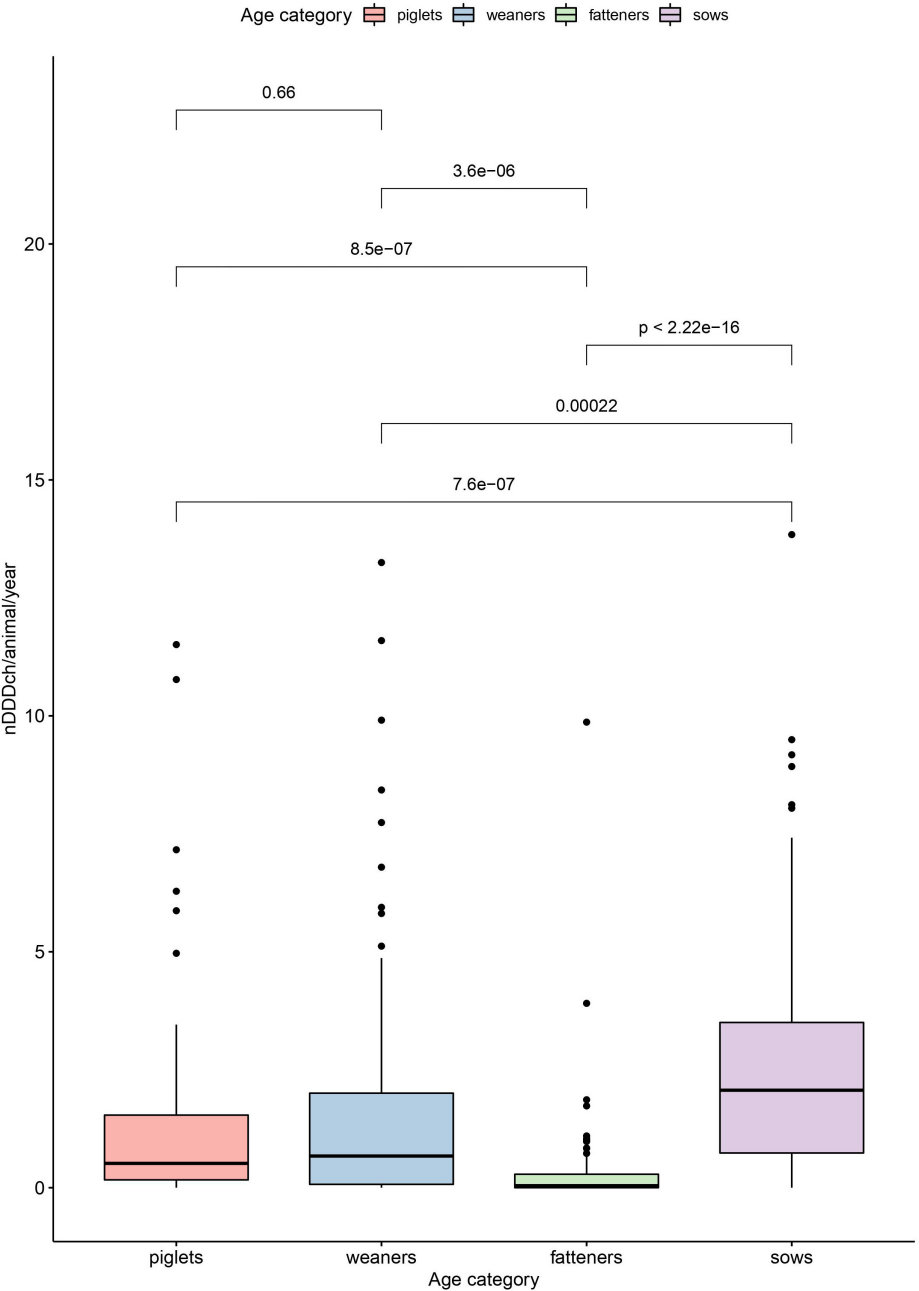
Boxplot showing differences in the number of defined daily doses Switzerland per pig (nDDDch/animal/year) among the individual age categories (piglets, weaners, fatteners, and sows) investigated by a Kruskal-Wallis test followed by a *post hoc* pairwise analysis (Bonferroni correction).

The results of these calculations for nDDDch/animal/year values in the different age categories as well as the overall AMU per animal on the farms are summarized in Table 3 and visualized in Figure 2. Based on the defined daily doses, a significantly higher AMU per pig was found for sows (median of 2.1 nDDDch/animal/year) compared to the other age categories, whereas a significantly lower AMU was observed for fatteners (median of 0.04 nDDDch/animal/year) (Figure 2). No significant differences were observed between piglets and weaners, as indicated by their median values of 0.5 and 0.7 nDDDch/animal/year, respectively. The maximum values of nDDDch/animal/year on individual farms varied between 9.9 for the fatteners and 13.8 for the sows.

**Table 3:** Median values, minimum, and maximum and 25%/75% quartiles of the number of defined daily doses Switzerland per pig (nDDDch/animal/year) measured on 71 farrow-to-finish farms in the year 2017.

	**Median**	**Minimum**	**25% Quartiles**	**75% Quartiles**	**Maximum**
1. Piglets	0.5	0	0.2	1.5	11.5
2. Weaners	0.7	0	0.1	2.0	13.3
3. Fatteners	0.04^*^	0	0	0.3	9.9
4. Sows	2.1^+^	0	0.7	3.5	13.8
Overall	5.2	0	3.0	8.1	26.1

* *P < 0.001 to 1, 2, and 4*;

+ *P < 0.001 to 1, 2, and 3 by a Kruskal-Wallis test followed by a post-hoc pairwise analysis (Bonferroni correction)*.

### Relationship of AMU Among Age Categories

**Figure 3:**
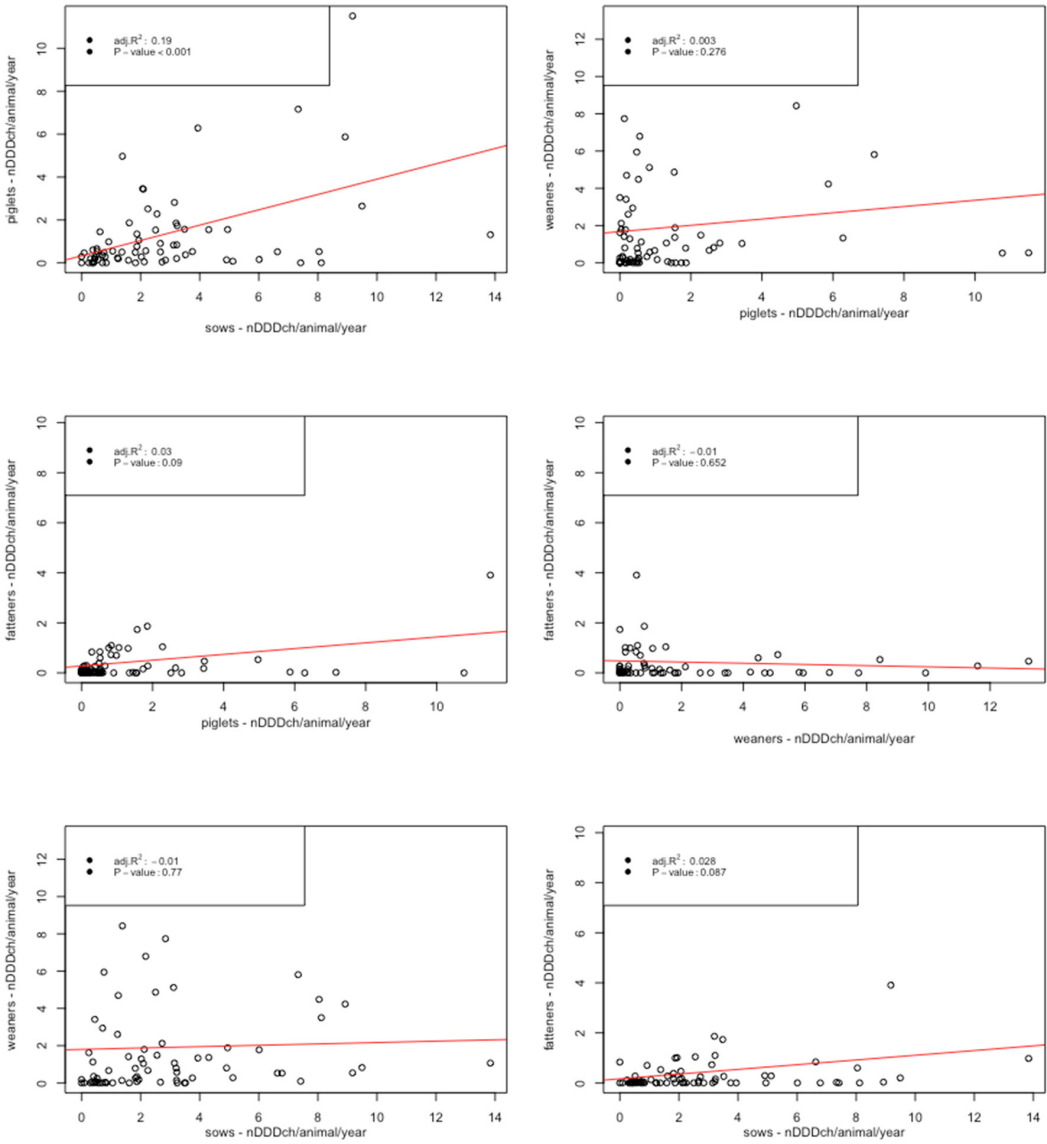
Scatterplots and linear regressions for the within-herd relationship of the number of defined daily dose Switzerland per pig (nDDDch/animal/year) among the different age categories (piglets, weaners, fatteners, and sows) for 71 farrow-to-finish pig herds in Switzerland.

Considering the within-herd AMU of the different age categories measured as defined daily doses per animal and year, a significant linear relationship was found between data for sows and piglets (*P* < 0.001) (Figure 3). The analysis of other age category combinations showed some positive (e.g., piglets and weaners) and some negative (e.g., weaners and fatteners) relationships—none of them significant.

### AMU Quantification per Herd Size

**Figure 4:**
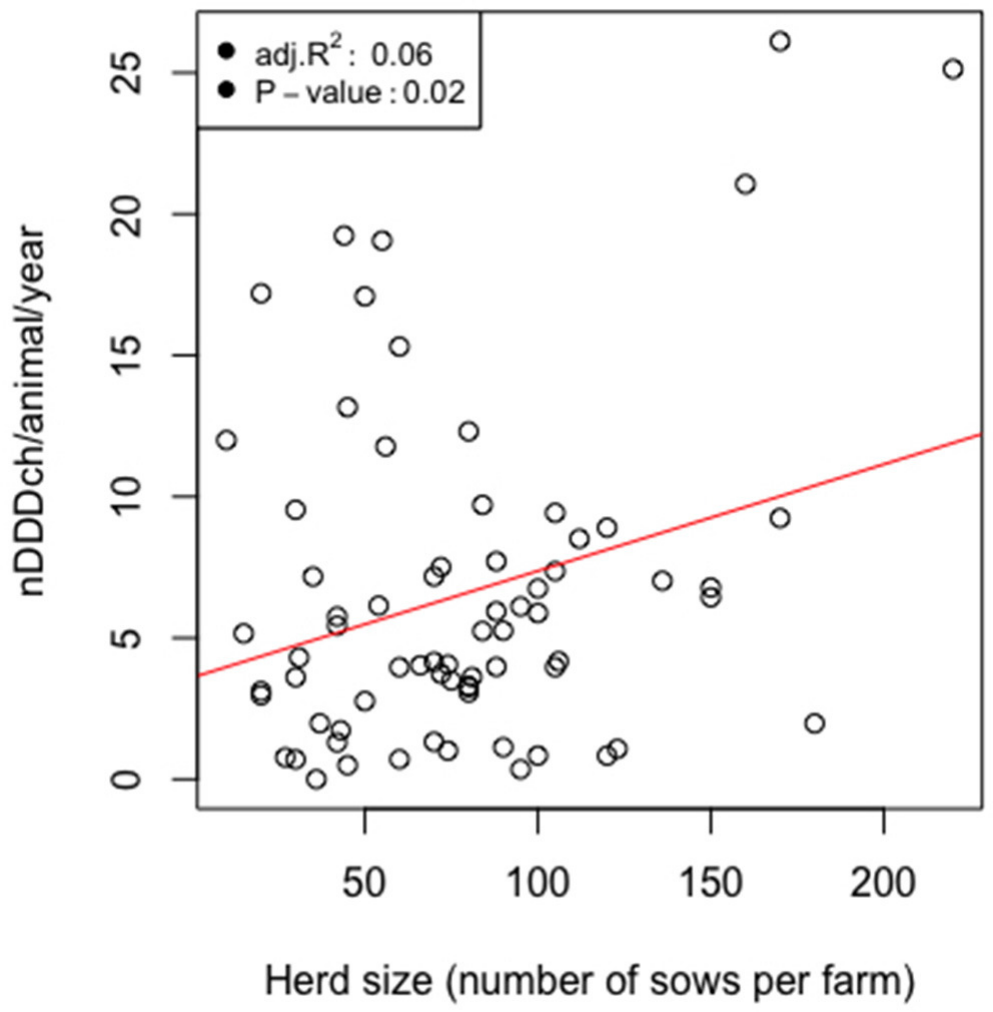
Scatterplots and linear regressions for the relationship of the number of defined daily dose Switzerland per pig (nDDDch/animal/year) and the herd size counted as the number of kept sows for 71 farrow-to-finish pig herds in Switzerland in 2017.

As shown in Table 2, the average, minimal, and maximal herd size of our study population measured as kept sows were 10, 79, and 220 animals per farm, respectively. The median value of antimicrobial drug usage was 5.2 defined daily doses per animal per year and reached a maximum of 26.1 on one farm (Table 3). Figure 4 shows that both variables were connected by a significant linear relationship between the herd size measured as kept sows and the AMU measured as defined daily doses per animal and year (*P* = 0.02).

## Discussion

This study found major differences in AMU among the age categories of piglets, weaners, fatteners, and sows, which varied depending on whether active ingredients or the number of defined daily doses are used as method of measurement. Due to the different weights of the age categories, 9.8 kg (10%) of all active ingredients in piglets represent 50% of the number of defined daily doses, whereas 36.1 kg (36%) in sows represents only 3% of the total number of these doses. For the weaners, the percentage for both the active ingredients and the number of defined daily doses was 44%. As previous studies have shown, the proportion of AMU measured by defined daily doses in weaners is comparatively high (18, 34). A frequent administration of antimicrobial premixes contained in the feed for a certain period in this age category has also been documented (35). In Switzerland, some of the available premixes include high concentrations of active ingredients (23), which, combined with relatively low animal weight and longer treatment duration, could explain the results obtained for weaners.

This could also partly explain that in weaners, in contrast to the other age categories, it is not penicillins, often used as an injection solutions, but sulfonamides and tetracyclines that were the most frequently observed antimicrobial classes within the amount of active ingredients. Both sulfonamides and tetracyclines are potentially part of the premixes, which are used as group treatments in weaners in Switzerland (36). This study, similarly to other Swiss (22, 37) and international studies (18, 38), observed penicillin as a frequently used antimicrobial class in the different age categories of pigs. Since penicillins are considered as the first selection for many indications in the Swiss AMU guidelines for pigs, an evidence-based usage in accordance with guidelines could be concluded from this findings (8).

This study shows that for HPCIAs, the calculated number of defined daily doses was 21% of the overall consumption. Similar results with similar measuring methods have already been shown in earlier studies, which could also demonstrate the potential for reduction over time (10, 39). Similarly to the deviations observed for the different age categories, the lower percentage of 7% HPCIAs measured as active ingredients shows the differences in output between the two measurement methods of defined daily doses and amount of active ingredients.

These differences in the output of different measurement methods were mentioned in theoretical reviews (14, 40) and observed in field trials (24, 41). They also need to be considered, for example, when evaluating comparisons of the AMU among countries or species. In principle, monitoring systems based on defined doses only allow a statistical estimation of the probable AMU since the calculation is based on prescribed amounts and the doses used on the farm could differ from the estimations. Other authors therefore prefer the so-called “used daily doses” if detailed data about the on-farm doses of individual treatments are available (42). However, the calculation behind the number of doses per animal and year as described in this study is comparable to other systems using defined doses to estimate AMU in livestock (43, 44).

The average herd size of the farms in this study was 79 kept sows, which is comparable to earlier studies describing the usage of antimicrobials on Swiss pig farms (36). Since participation in the Suissano/Safety+ program is voluntary, farmers with a higher awareness of the importance of antimicrobial treatments in their animals and the role it plays in the spread of resistance maybe more represented (45). This might result in a selection bias in the study population and could compromise the internal validity of the analyses in this study. Although participation in the program is without benchmarking or consequences, a reduction of AMU on farms could be observed as a result of the comparison to other farms (39).

This study identified significant differences in the number of defined daily doses per animal among the different age categories. Fatteners were treated less frequently, which has already been shown in a previous study (18) and could be explained by the good health status of Swiss pigs in terms of lung diseases (46) as well as a low number of oral group therapies with certain combinations of antimicrobials compared to weaners (22). As Switzerland is almost free of the lung pathogens *Mycoplasma hyopneumoniae* and *Actinobacillus pleuropneumoniae* and free of the porcine reproductive and respiratory syndrome (PRRS) virus, there are some indications missing which have been linked to increased antibiomicrobial usage in growing pigs in other countries (13, 47). The continuous development of the Suissano/Safety+ program will allow the identification of indications for treatment as well as management and biosecurity measurements on Swiss pig farms and the evaluation of these potential confounders on the AMU will be the subject of future studies. For the present study, confounders were minimized by restricted sampling that included only farms with a complete AMU data documentation of the year 2017 into the study (48).

In contrast to the lower AMU in fatteners, the AMU in sows measured as defined daily doses per animal was significantly higher compared to all other age categories. This finding was observed also by other authors (24, 38), although other studies refute it (18, 26). One of the main drivers of high AMU in sows could be the treatment of postpartum dysgalactic syndrome (PPDS) after birth. PPDS has been described as an important cause of economic losses in sows (49) and Pendl et al. (50) were able to show the potential of reducing AMU by targeted herd health management for this syndrome. Since PPDS is related to reduced colostrum production, it could also partly explain the significant relationship between the AMU of sows and suckling pigs identified in this study. The connection between reduced colostrum intake and increased mortality and susceptibility to disease such as diarrhea caused by *Escherichia coli* has been observed previously (51, 52); increased usage of antimicrobials for piglets in such cases could therefore also be assumed. In contrast to Sarrazin et al. (18) and Sjölund et al. (26), this was the only significant relationship among the different age categories in this study. Although there were some positive (e.g., piglets and weaners) and negative (e.g., weaners and fatteners) relationships between the other age categories, none of them were significant. These findings therefore indicate the importance of stratified AMU monitoring which evaluates the results of the different age categories separately and not as an overall farm assessment. The benefit of such a monitoring system is also underlined by the fact that the AMU in single farms reached high maximum values in individual age categories. This is already implemented in the Suissano/Safety+ program.

Similarly, a significant relationship between larger herd size measured as kept sows and increased AMU was observed. This underlines the findings of previous studies (27, 28) and emphasize the importance of successful herd management and appropriate external and internal biosecurity practices to avoid disease spreading and maintain standards of animal health and low antimicrobial consumption, especially on larger farms. To continue understanding the influence of various farm characteristics on AMU as well as important indications, as has been done in other studies (29), further research is needed for the Swiss pig sector.

## Conclusion

Our study demonstrates that AMU monitoring programs should evaluate different age categories separately to identify specific points of concern and suggest possible solutions for individual farms. The categories of piglets and sows are especially important on Swiss pig farms due to their high and related AMU either in absolute terms or per animal. Likewise, a link between larger farms and increased usage was observed and should be considered through improved on-farm health management.

## Data Availability Statement

The raw data supporting the conclusions of this article will be made available by the authors, without undue reservation, to any qualified researcher.

## Ethics Statement

Ethical review and approval was not required for the animal study because since this was only data that had no influence on the actual treatment of the animals, an animal welfare permit was not required. No veterinary manipulations or similar interventions were carried out on any animals by the research team. Written informed consent was obtained from the owners for the participation of their animals in this study.

## Author Contributions

TE drafted the manuscript. TE, DK, and XS designed and directed the study. CM supported the pharmacological aspects of the used measurement methods. TE prepared the data processing and statistical analysis. All authors have read and approved the manuscript.

## Conflict of Interest

The authors declare that the research was conducted in the absence of any commercial or financial relationships that could be construed as a potential conflict of interest.
